# Ensuring Food Integrity by Metrology and FAIR Data Principles

**DOI:** 10.3389/fchem.2018.00049

**Published:** 2018-05-22

**Authors:** Michael Rychlik, Giovanna Zappa, Larraitz Añorga, Nastasia Belc, Isabel Castanheira, Olivier F. X. Donard, Lenka Kouřimská, Nives Ogrinc, Marga C. Ocké, Karl Presser, Claudia Zoani

**Affiliations:** ^1^Chair of Analytical Food Chemistry, Technical University of Munich, Freising, Germany; ^2^Centre for Nutrition and Food Sciences, Queensland Alliance for Agriculture and Food Innovation, University of Queensland, Coopers Plains, QLD, Australia; ^3^ENEA, Italian National Agency for New Technologies, Energy and Sustainable Economic Development, Department for Sustainability of Production and Territorial Systems, Biotechnologies and Agro-Industry Division, Casaccia Research Center, Rome, Italy; ^4^CIDETEC, Centre for Electrochemical Technologies, Parque Científico y Tecnológico de Gipuzkoa, San Sebastián, Spain; ^5^National R&D Institute for Food Bioresources, IBA Bucharest, Bucharest, Romania; ^6^Department of Food and Nutrition, National Health Institute Doutor Ricardo Jorge, Lisbon, Portugal; ^7^IPREM, Institut des Sciences Analytiques et de Physicochimie pour l'Environnement et les Matériaux, Université de Pau et des Pays de l'Adour, Pau, France; ^8^Department of Microbiology, Nutrition and Dietetics, Faculty of Agrobiology, Food and Natural Resources, Czech University of Life Sciences Prague, Prague, Czechia; ^9^Dept. of Environmental Sciences, Jožef Stefan Institute, Ljubljana, Slovenia; ^10^National Institute for Public Health and the Environment, Bilthoven, Netherlands; ^11^Premotec GmbH, Winterthur, Switzerland; ^12^Department of Computer Science, ETH Zurich, Zurich, Switzerland

**Keywords:** food authenticity, food fraud, food safety, METROFOOD-RI, metrological traceability, reference materials, research infrastructures, Horizon 2020

## Abstract

Food integrity is a general term for sound, nutritive, healthy, tasty, safe, authentic, traceable, as well as ethically, safely, environment-friendly, and sustainably produced foods. In order to verify these properties, analytical methods with a higher degree of accuracy, sensitivity, standardization and harmonization and a harmonized system for their application in analytical laboratories are required. In this view, metrology offers the opportunity to achieve these goals. In this perspective article the current global challenges in food analysis and the principles of metrology to fill these gaps are presented. Therefore, the pan-European project METROFOOD-RI within the framework of the European Strategy Forum on Research Infrastructures (ESFRI) was developed to establish a strategy to allow reliable and comparable analytical measurements in foods along the whole process line starting from primary producers until consumers and to make all data *findable, accessible, interoperable, and re-usable* according to the FAIR data principles. The initiative currently consists of 48 partners from 18 European Countries and concluded its “Early Phase” as research infrastructure by organizing its future structure and presenting a proof of concept by preparing, distributing and comprehensively analyzing three candidate Reference Materials (rice grain, rice flour, and oyster tissue) and establishing a system how to compile, process, and store the generated data and how to exchange, compare them and make them accessible in data bases.

## Introducing food integrity

Consumers increasingly demand foods of high “integrity,” which is a comprehensive term for sound, nutritive, healthy, tasty, safe, authentic, traceable, as well as ethically, safely, environment-friendly, and sustainably produced foods (Elliott, 2012). In particular, food chain integrity is a multidisciplinary issue covering all aspects of the food chain from producers to consumers and is based on chemical and microbial food safety, authenticity of food origin, and nutritional quality. To verify integrity in marketed products, two approaches are currently deemed most suitable: first is to maintain an inalterable line of traceability such as the block chain concept (storage and networking of information in a virtual open space as decentralized shared ledger), second the rigorous testing of the products under question by modern analytical methods. The tested whole set of analytes may also be summarized by the currently introduced term “foodome,” i.e., the “collection of all compounds present at a given time in an investigated food sample and/or in a biological system interacting with the investigated food” (Rychlik et al., 2017). This perspective communication will focus on challenges and visions for the latter approach including the benefits of applying metrology to food.

For assessing the foodome, a variety of analytical platforms may be applied, which all are commonly assigned to different “omics” methodologies, such as genomics, proteomics, metabolomics, metallomics, and isotopolomics (Rychlik et al., 2017).

These platforms can be differentiated into targeted and non-targeted variants, the latter of which provide a rather comprehensive picture of food components. However, targeted methods show, in general, supreme sensitivity for trace contaminants coming from raw materials, as we recently reviewed for mycotoxins (Rychlik et al., 2017) or for acrylamide coming from heating processes, for which maximum limits (MLs) are currently in place. Superior sensitivity is generally required for controlling ML set up to ensure food safety in the European Union.

Apart from food safety, the authenticity of foods is an increasingly important topic in analytical Food Chemistry. There is a growing consumers' awareness of fraudulent food practices, particularly in food categories like wine, meat, olive oil, spices, milk products, fish, honey, coffee, tea, and juices being on the hit list of adulterated products (European Commission, 2016a). The mislabelling, misbranding, or misrepresentation of food and food ingredients, or food packaging, or false or misleading statements made about a product, lead to decreasing of consumer's trust in food industry. A recent example is the 2013 horse meat scandal, in which foods labeled as containing beef were found to contain undeclared horse meat as evidenced by detecting horse DNA (Food Safety Authority of Ireland, 2018).

Another important aspect of food integrity is nutrient profile. Nutrients have gained significant relevance as protective or harmful factors and the World Health Organization (WHO), governments and the scientific community are making great endeavors to unravel the effect of food components on health and disease, and to improve the composition of foods regarding the content of sugar, salt, and fatty acids (WHO, 2015). Comparable, accurate and traceable analytical values in foods are necessary to guarantee a true estimation of nutrient intake and to monitor the populations' nutrient status at a national and European level over time (Westenbrink et al., 2016). In this context, labeling of nutrients is also an important tool for consumer education and healthy choices (Hieke et al., 2016).

Several research projects have concluded that accurate nutrient values at multi-center studies are difficult to compare if data collection of nutrient intake and food consumption is not done in a harmonized way. One of the pitfalls of these studies is the gap in metrological tools observed in measurements at European level when multi-center studies were launched (Van Schoor et al., 2017). WHO and EFSA have highlighted a number of limitations, including the lack of robust quality assurances procedures in laboratories generating analytical data (FAO, 2011).

For addressing all these issues and generally in food analysis, sensitive, specific, fully validated, standardized, and officially authorized methods of superior quality are essential. For quality assurance, two approaches may be followed, the first involving Proficiency Testings (PTs), Interlaboratory, and Round Robin trials. The second approach involves the use of Reference Materials (RMs) tested for stability and homogeneity with certified, reference or information values for the properties under study. These values ideally are generated by reference methods with superior accuracy and/or reference laboratories.

## The concepts of metrology applied to foods

In food analysis generally there is the need to introduce the metrological concepts into chemical and biological measurements. Based on the definition of metrology as “the science of measurement, embracing both experimental and theoretical determinations at any level of uncertainty in any field of science and technology” (BIPM Bureau International des Poids et Mesures, 2017), its concepts include the definition and realization of internationally accepted units of measurement and the (metrological) traceability by assessing uncertainty in relation to national and international reference standards measurements. Moreover, metrology provides the tools to make the measurement results reliable and comparable. Quality of measurements plays increasingly a key role in the technological and socio-economic development, thus supporting trade and quality demonstration of products and services, and strengthens the knowledge base for decision-making in the environmental, health, and forensic sectors. In food analysis RMs play an important role in adopting metrological concepts.

Currently available RMs are certified for a rather limited number of parameters, and for rather limited matrixes, which makes them usable only for a restricted number of methods. The need for new RMs is closely related to the new analytical needs and the emerging challenges of food safety (e.g., application of nanotechnologies or biotechnologies), or are needed to meet the new requirements related to the analytical determination of nutraceutical substances and natural substances with protective action on health. A particularly pressing need is linked to the implementation of new RMs to be employed for assuring origin and traceability of raw materials and products and for the detection of frauds and adulterations (Zappa and Zoani, 2015). In this view, new RMs are increasingly necessary to identify genetic markers and chemical profiles and to verify the geographical and/or biological (botanical, zoological, genetic) origin of raw materials and products. Particularly interesting, especially with reference to assess the foodome, is the possibility to prepare *Multiparameter*-RMs intended for use in multiparameteric determinations, qualitative analyses, and identity studies through the definition of elemental, isotopic, molecular, and/or genetic markers or patterns for traceability of food products (Zoani et al., 2014). In particular, novel analytical technologies and concepts such as non-targeted metabolomics using ultra-high resolution mass spectrometry (Rychlik et al., 2017) or inductively coupled plasma mass spectrometry with multicollection (ICP/MS MC) for multielemental inorganic and isotopological analysis (Drivelos and Georgiou, 2012) offer promising perspectives.

A more comprehensive characterization of the foodome present in these materials, other than to promote standardization on multiparametric characterisation, would be of additional value on the one hand, for laboratories to avoid the costs for acquiring different materials for the required set of analytes, and, on the other hand, for the RM producers, which can save efforts for example for the testing of homogeneity.

Complementary to the use of RMs, quality and traceability of measurement results have to be secured by an appropriate calibration of equipment, especially the critical items. Thus, the traceability chain defined as a sequence of standards and calibrations that form a link between a result of a measurement and a reference should be established and maintained.

Regarding the current situation in food analysis and research, a huge fragmentation in Europe and worldwide can be stated. Apart from the common European Commission's regulations on official food controls and common notifications within the EU Rapid Alert System for Food and Feed (RASFF), the general goal of verifying food integrity is approached in every European member state separately despite food production, marketing, and fraud occurring multi-nationally and even globally. Moreover, there is a huge variety of scientific disciplines and types of organizations involved. Even worse, the data outcome of these control and verification processes is entered and distributed in a vast variety of different databases. Many efforts appear to be redundant by duplication and gaps not to be systematically addressed.

Here, metrology leads to a systematically aligned common structure for measurements and compiling the results could create a common and shared base of data and knowledge.

## Advantages related to data sharing and integration in the food sector

New technologies are leading to an exponential increase in the volume and types of data available, creating unprecedented possibilities for generating new knowledge and leading to societal changes. Governments, economy, researchers, and citizen groups are in a process of experimentation, innovation, and adaptation to the new world of data, a world in which data are bigger, faster, and more detailed than ever before. Without high-quality data providing the right information on the right topics at the right time, it becomes almost impossible to design, monitor, and evaluate effective policies. Moreover, in dealing with the generation, handling and providing of data, it is fundamental to refer to the principles of FAIR data, i.e., to make the data *Findable, Accessible, Interoperable, and Re-usable*. Meeting these principles will lead to

- reduced costs (for producing and managing data);
- improvement of the data quality and of the demonstrability of high quality and integrity of the data;
- availability of reliable data in real-time;
- reduction in the time between monitoring and action;
- more scientific research re-using available databases and using data in new combinations.
- building of public trust in the data, and expansion of the people's ability to use them.

Data quality and integrity can be enhanced by establishing standards and recommendations for appropriate analytical methods starting with defining Standard Operation Procedures (SOP) for analyzing data up to collecting and implementing experts' and users' quality feedback. Such a digital quality framework will periodically check data quality requirements against the database and provide feedback to data curators and users (Presser, 2012). Data accuracy and timeliness are also known as dimensions (= key concept) in the area of data quality research.

Agri-food, like other industrial sectors, is undergoing a major digital transformation that is redesigning the profile of the sector characterized by huge variety, contrasts, and segmentation. The real challenge for the agri-food sector is to realize a digital revolution even for small enterprises in order to fully integrate them into the change in progress (European Commission, 2016b). The increasing availability of data represents incredible potential resources to develop applications, models and systems that help different classes of users (consumers, entrepreneurs, policymakers) to make conscious decisions on quantitative and, above all, objective bases. The collection and transformation of more and more data into strategic and useful information is, however, a process that must be accompanied, on the one hand, by the growth of capabilities in fully exploiting the generated information, and on the other hand, by an increasingly shared approach. The latter can allow to overcome the intrinsic difficulties faced by the agri-food sector due to the segmentation and fragmentation of the enterprises, thus favoring not only individual growth but also aggregation of capabilities in consortia, districts, or business networks paving the way to de-centralized innovative governance models by means of *Distributed Ledgers*. In order to make the product path from farm to fork data more transparent, sharing, and integration could permit new approaches to traceability, as well as offer solutions to the emerging issues of Food Defense or, more generally, of Food Protection. Finally, the availability of Open Access Data platforms could permit the realization of functional application tools for collaborative sharing and validation of technological solutions.

## Perspective of a new research infrastructure

Current European food traceability systems lack the linkage of food chains records at the European level (Charlebois, 2014). Further pitfalls are the inaccuracy and errors in measurements as well as recording and delays in obtaining essential data, which is fundamental for quick and appropriate decisions, e.g., in case of outbreaks of food disease. Partly due to the multidisciplinary approach and multi-dimensionality, this research sector suffers also from a high fragmentation and dispersion of data, so it is essential to develop areas of common data to which the researchers from different areas can freely access in order to achieve integration, exchange and sharing. The need for measurement results traceable to the International System of Units and comparable worldwide is of concern to analytical laboratories, government authorities, industries and the public. Besides the European Metrological Infrastructure consisting of the National Metrology Institutes (NMIs) and EURAMET, the EU Joint Research Centre—Directorate F, WELMEC, and European co-operation for Accreditation (EA), and the Standardisation Bodies (ISO, CEN), there is a urgent need to realize coordination, integration, and synergies especially for Applied Metrology and namely Metrology in Food and Nutrition. Despite the wide availability—in some cases—of official methods, the chemical and biological measurement lack evidence of a proven metrological linkage and are still open to improvements in analytical reliability by applying metrological principles. Furthermore, the access to official methods in many cases is not free (i.e., CEN or ISO methods, AOAC—http://www.aoac.org—and ASTM—http://www.astm.org—methods) and for most of the chemical and biological measurements, reference methods and fit for purpose RMs are not available yet. Some databases list the available PTs organized by the different providers, such as the BIPM KCDB (*Key Comparison Database*—https://kcdb.bipm.org) and the EPTIS database (*European Proficiency Testing Information System*—http://www.eptis.org). The same applies for RMs, for which some databases on the worldwide production are available on line (e.g., COMAR, IAEA, *Japanese RMs search*, VIRM) but they cover a huge variety of components (not specific for the food sector) and are designed for including any kind of RM. As a consequence, searching of RMs for a specific application field—and especially for the food sector—is often difficult and distracting.

In order to generate more effective European food chain systems, to integrate and harmonize scientific research in the field of food quality and safety, and to promote metrology for food and nutrition, a new Research Infrastructure (RI) titled “*Infrastructure for promoting Metrology in Food and Nutrition”* (METROFOOD-RI, www.metrofood.eu) is being developed in the frame of the European Commission's Research Strategy.

With the aim to integrate METROFOOD-RI into the European landscape, to fill the gaps of the existing RIs, to prevent duplications and to identify the future trends, an in-depth analysis of the pan-European landscape on RIs at EU level has been performed. In particular, starting from the classification of European Strategy Forum on Research Infrastructures (2017) scientific domains, first of all the “Health & Food” domain has been considered as the main scientific one to which METROFOOD-RI belongs, besides to the “Environment” one, in which for some aspects the scientific case of METROFOOD-RI falls also.

In total over 140 RIs and networks at National and EU level related to Food, Health & Environment have been identified. The performed Landscape Analysis showed that there are some significant gaps in pan-European RIs and, therefore, the strong need to develop and link new RIs on “Food & Nutrition”, as well as on “Sustainable Agriculture & Bioeconomy” in the complex medical and agriculture fields. In this context, METROFOOD-RI will cover these gaps by providing high quality metrology services in food and nutrition.

This RI will allow for coordination of the goals mentioned above on a European scale and opening scenarios on a global scale. Thus, duplication of efforts is avoided and coordination and harmonization are promoted. The general objective is to enhance scientific cooperation and encourage interaction among the various stakeholders, as well as the creation of a common and shared base of data, information and knowledge. Including metrology, METROFOOD-RI embraces both experimental and theoretical determinations and is characterized by a holistic approach to the agri-food sector, which implies measurements being carried out from primary production until final consumption all along the supply chain. As an important goal, primary producers should be enabled to better understand the analytical data, and analysts should be better informed about the way the raw plant and vegetable materials are produced and which factors could affect it. All of them should also consider the changes of the foodstuff during the harvesting, processing, and storage. Finally, the consumers should be enabled to better understand the information about the food composition (analytical data) given to them via media and other sources.

METROFOOD-RI combines a physical distributed infrastructure (p-RI) and a virtual one (e-RI), and is intended to provide distributed services, acting on the real plan of measurement reliability and procedure harmonization and adopting the FAIR principles on data management and provision of e-services. METROFOOD-RI will act in a dynamic way as its services will be in real time adapted to the stakeholder's needs in order to offer the best measurement results and solutions for keeping an appropriate food quality. The main target audience for the dissemination of METROFOOD-RI outputs will be the research community, food business operators and economic players, policymakers/food inspection and control agencies, consumers/citizens and media.

This pan-European initiative, coordinated by the Italian National Agency for New Technologies, Energy and Sustainable Economic Development (ENEA), currently involves 48 partners from 18 European Countries. In each Country, METROFOOD-RI is being organized in National Nodes and some dedicated Joint Research Units have been established (e.g., in Italy, FYROM, Greece, Portugal, and Slovenia). The services METROFOOD-RI intends to provide include:

- metrological and standardization services (big-scale production of RMs, development of new methods and devices, provision of PTs, etc.);
- the offer of exceptional analytical/diagnostic capacities for the entire production chain;
- services aimed at improving food production and consumption;
- advanced training for academics and professionals (B.Sc., M.Sc., Ph.D. students, researchers, academics from research institutions, analytical laboratory staff, consumers/citizens, food chain quality managers, food company staff);
- e-services for measurement standardization and harmonization, food production and processing, as well as for access to food data, data linkage, and data analysis.

**Figure 1:**
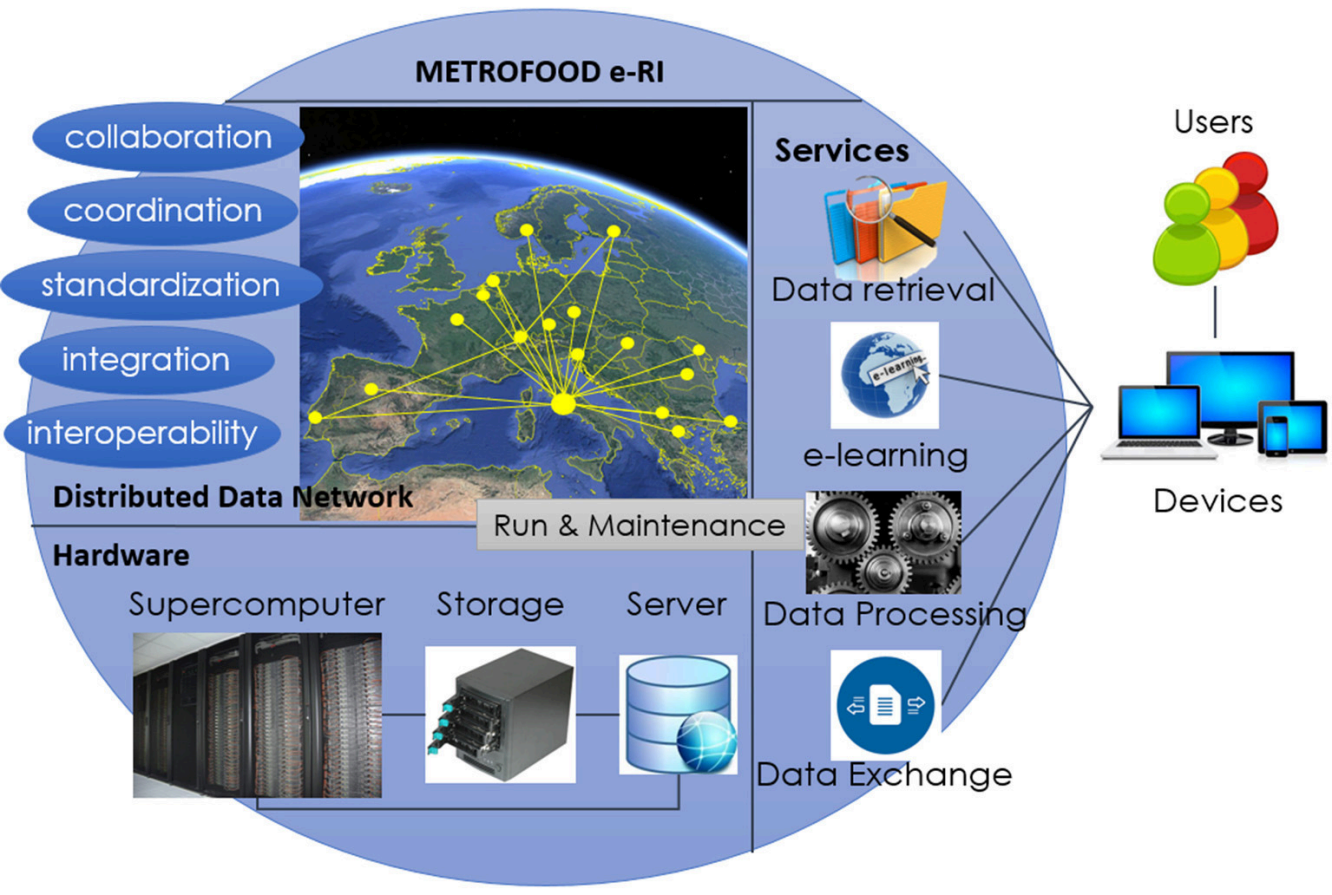
Basic building blocks of the METROFOOD e-RI with data network, hardware, and service layer.

The METROFOOD e-RI can be considered as a network of interlinked data providers, which is encapsulated as one European-wide data source for users as shown in Figure 1. The e-RI provides services for data retrieval from multiple sources, linkage of data, tools to operate on data as well as knowledge bases. Such an e-RI requires from data providers (laboratories) satisfying minimum performances (e.g., SI traceability, LOD, LOQ, reproducibility), data collection and data management procedures to ensure standardization and harmonization of data formats and interfaces, and a continuous data quality and provenance evaluation.

In particular the combination of data related to food safety will allow new approaches for risk and risk-benefit assessments. The e-RI will collect food consumption data and, combining them with the collected data on contents of contaminants, will enable a comprehensive exposure assessment with information on uncertainties included in the data sources (e.g., from metrological aspects). The generated exposure data will be related to health-based guidance values (e.g., Tolerable Daily Intake) or, if these are lacking, to the Threshold of Toxicological Concern (TTC) concept for risk assessment. Even more importantly, METROFOOD-RI will collect data for contaminants and for beneficial compounds simultaneously. Thus, risk-benefit assessments for adversely and protective bioactive compounds within one food (e.g., methylmercury vs. omega-3-fatty acids in seafood, Hoekstra et al., 2013) as well as for compounds with both protective as well as adverse bioactivities (e.g., folic acid in fortified foods, Hoekstra et al., 2008) will be possible.

METROFOOD-RI has now completed its “Early Phase” funded within the EU Horizon 2020 scheme as the PRO-METROFOOD project (INFRADEV-02-2016 GA n. 739568) and has recently applied for inclusion in the ESFRI Roadmap 2018 as “Active” Project, so to approach the next phases (Preparatory Phase, Implementation, and Operation). In 2017 (Early Phase) the RI devised its future structure and organization, as well as the portfolio of services, defined the plans and strategies for its long term sustainability and the plans for training, communication, dissemination and exploitation, and provided a proof-of-concept by the development of two pilot services. One of these pilots referred to the development and provision of new RMs and involved the preparation and characterisation of three new RMs: rice flour, rice grains and lyophilized oyster tissue. Matrixes were selected with the aim to represent cereals as staple food of worldwide relevance and a food matrix of animal origin known to accumulate a huge variety of contaminants and residues (oysters). Further, these matrices are important for verifying authenticity of often adulterated products. In order to finally promote the production of *Multipurpose*-RMs and test the capability to perform a comprehensive RM characterization, many different parameters were considered within the pilot study during the PRO-METROFOOD project (Table 1). Parameters were selected based on the possibility to involve in the characterisation as many laboratories of the Consortium as possible and perform cross-comparisons, with the final purpose to test and demonstrate the inter-operability of the infrastructure. On the whole, the pilot allowed to better set up and organize the specific service selected, to demonstrate the actual capability of METROFOOD-RI to deliver services and to test its inter-operability. Participating laboratories have been enabled to assess their performance relative to domestic and international peer laboratories and hence to improve the comparability of results among laboratories and among Countries. Specifically concerning these RMs, the next steps involve the completion of homogeneity and stability testing, the improvement of the characterisation focusing on some specific parameters missing in currently available RMs for the same matrixes and on food safety issues related to the specific matrixes and then to obtain the certification for some selected parameters. In this way, such (certified) RMs will be made available for the market. This will allow also to further improve the capability of the Consortium to provide such kind of services (provision of new—customized—RMs, organization and management of PTs) and actually allow the access of external users to the services themselves.

**Table 1:** Parameters considered for characterization of the reference materials produced during the pilot service implemented in PRO-METROFOOD.

**Characterised Parameter**		**Applied Methods**
**Nutrients and bioactive compounds**	Sugars, carbohydrates	HPLC-RI, Ewers method
	Fibers (total, crude, dietetic; amylose content)	Enzymatic method, Henneberg-Stohmann method, Flow Injection Spectrophotometric Analysis
	Mineral salts (Ca, K, Na, Mg, P) and trace elements	AAS, ICP-AES, ICP-MS
	Vitamins (C, B group)	LC-MS/MS
	Lipids	Extractive gravimetry
	Fatty acids	GC-FID
	Protein fractions	Kjeldahl method, LC-MS/MS, Spectroscopy (Bradford)
	Amino acids	UPLC-PDA
	Phenols	Spectrophotometric analysis, HPLC, LC-MS/MS
	Tocols	HPLC-DAD
	Flavonoids and flavonols	Spectrophotometric analysis
**Bioactivity and reactivity**	Antioxidant activity	Fluorimetric analysis, UV-Vis Analysis, DPPH scavenging assay
**Organic Contaminants and Residues**	Toxigenic fungi and toxins	LC-MS/MS, HPLC-FLD, ELISA
	Pesticides, pharmaceutical residues and veterinary drugs	GC-MS, GC-ECD, LC-MS
**Inorganic Contaminants**	Toxic elements (e.g., As, Cd, Cu, Hg, Ni, Pb)	ET-AAS, AMA, ICP-AES, ICP-MS, ID-ICP-MS, MC-ICP-MS, TXRF, INAA
	Speciation (As speciation, Hg and MeHg, Sn, and organotin compounds)	HPLC-ICP-MS, GC-ICP-MS
**Isotopes** (e.g., Hg and Sr isotopes, stable isotope analysis of light elements)		MC-ICP-MS, EA-IRMS
**Microbiological analysis** (e.g., membrane filtration; occurrence of *Campylobacter jejuni*, C. coli, enterohemorrhagic *Escherichia coli, Salmonella* spp., *Vibrio parahaemolyticus, V. vulnificus*, emetic type of food-borne *Bacillus Cereus* species group)		Membrane filtration, accumulation techniques, fluorescence
**Physical characterization** (e.g., ash, particle size, gelatinization-retrogradation, texture parameter of cooked rice)		Gravimetry for ash; transmission electronmicroscopy, scanning electronmicroscopy for particle size; differential scanning calorimetry for gelatinization-retrogradation
**Allergen profile characterization**		Gel electrophoresis, LC-MS/MS characterization and protein identification by advanced bioinformatic tools
**Genetic Analyses**	Genotyping	Feedcode TBP (Tubulin Based Polymorphism)
	Rice endogenous/GM construct screening^*^	PCR

* *only for rice grains and rice flour*.

This pan-European initiative based on the application of metrology may effectively contribute to the overall societal challenge of authentic and safe food worldwide, working to align research and innovation with the values, needs and expectations of the society in accordance with the European Responsible Research and Innovation (RRI) principles.

## Author contributions

All authors listed have made a substantial, direct and intellectual contribution to the work, and approved it for publication.

### Conflict of interest statement

The authors declare that the research was conducted in the absence of any commercial or financial relationships that could be construed as a potential conflict of interest.
